# Influence of repeated mastitis on the milk production and metabolic status in the subsequent lactation period of dairy cattle

**DOI:** 10.1016/j.heliyon.2024.e29789

**Published:** 2024-04-18

**Authors:** Shiho Miyata, Lijie Fan, Jun Kambe, Mohammad Ibrahim Qasimi, Satoshi Takemoto, Masahiko Ito, Chunmei Li, Yuki Yamamoto, Kentaro Nagaoka

**Affiliations:** a Laboratory of Veterinary Physiology, Department of Veterinary Medicine, Tokyo University of Agriculture and Technology, Tokyo, 183-8509, Japan; b Central Research Institute for Feed and Livestock, ZEN-NOH (National Federation of Agricultural Cooperative Associations), Ibaraki, 319-0205, Japan; c Department of Virology and Parasitology, Hamamatsu University School of Medicine, Shizuoka, 431-3192, Japan; d College of Animal Science and Technology, Nanjing Agricultural University, Nanjing, China

**Keywords:** Repeated mastitis, Dairy cow, Metabolites, Biomarker

## Abstract

Recurrent mastitis poses a common challenge on dairy farms. While the impact of repeated mastitis within the same lactation has been investigated, the difference from one lactation to the next, particularly concerning the change of milk and blood metabolites, remains unclear. This study aimed to examine the difference in milk yield, milk composition, and metabolic status in the subsequent lactation between healthy and repeated mastitis in the previous lactation. The study population comprised 50 cows chosen from 400 cows, with 25 having no history of mastitis and 25 experiencing mastitis more than three times during the last lactation. Following dry-off and calving, all cows initiated a new lactation, during which no mastitis was diagnosed until the sample collection period. In the group exposed to repeated mastitis, a significant decrease in milk fat levels was observed in the subsequent lactation, while no change was observed in milk somatic cell count (SCC). Milk collected from cows that had experienced repeated mastitis in the previous lactation exhibited significant increases in the levels of free amino acids, namely valine, proline, and alanine. However, no difference in plasma levels of these amino acids was noted. These results indicate that individuals exposed to repeated mastitis have persistent milk quality changes even after dry-off. Biomarker analysis suggested that the milk valine and proline showed a moderate biomarker potential on Kappa coefficients to characterize cows that have experienced repeated mastitis. Furthermore, the results of biomarker combinations for valine and proline provided the highest specificity (100 %), positive likelihood ratio (infinity), and substantial biomarker potential on kappa coefficients (0.68). These findings significantly enhance our understanding of the pathobiology and etiology of recurrent mastitis and provide a biomarker to characterize cows that have experienced repeated mastitis in the past.

## 1 Introduction

Mastitis, an inflammatory condition, is the most prevalent disease in dairy cattle. It leads to reduced milk yield and quality, increased treatment costs, and additional labor activities, resulting in substantial economic losses for the global dairy industry [[1], [2], [3], [4], [5]]. It is estimated that approximately 72.8 % of cows exposed to clinical mastitis recover, while the remaining 24 % are culled or sold due to poor lactation and milk quality problems [6,7]. When pathogenic bacteria invade the mammary gland, injured mammary cells release cytokines, attracting leucocytes, particularly polymorphonuclear neutrophils (PMN), from the bloodstream into the milk. The leukocyte, which secretes substances to combat the bacteria, is the main milk somatic cell count (SCC) [[8], [9], [10]]. Mastitis diagnosis often involves detecting an elevated SCC, strongly associated with alterations in milk's protein quality, fatty acid composition, lactose, and mineral content [11]. Additionally, several studies have demonstrated changes in the milk of healthy adjacent quarters when a single quarter is affected by mastitis [[12], [13], [14]]. This suggests that mastitis pathogens directly affect mammary gland cells and impact adjacent glands by altering milk components.

Omics, encompassing genomics, proteomics, and metabolomics analyses, have been extensively used in biological research, including mastitis diagnosis [15]. Focusing on proteins and metabolites, which reflect cellular functions, is a practical approach for gaining insights into the body's metabolic status at any given time. Studies have revealed significantly higher levels of free amino acids and fatty acids in mastitis milk, attributed to increased proteolysis and lipolysis by neutrophils and plasmin from blood [11,17,18]. Casein degradation during mastitis leads to elevated levels of free amino acids, negatively affecting milk-derived products [11]. Furthermore, mastitis can increase the concentrations of free amino acids and organic acids in plasma, influencing the immune response and disrupting the Tricarboxylic Acid (TCA) cycle [15,[18], [19], [20]].

Recurrence of mastitis is a persistent issue associated with this disease. A high proportion of cows experiencing mastitis during the first 100 days of lactation are likely to experience additional mastitis episodes within the same lactation, and cows exposed to repeated mastitis often exhibit shorter lifespans and reduced production abilities [8,21,22]. It has been observed that the persistence of pathogens in the udder contributes significantly to the occurrence of repeated mastitis [23,24]. These phenomena are strongly associated with milk quality, and reduced profits due to milk quality changes also have a negative impact on dairy farmers [6,7,11]. Therefore, further examining the relationship between changes in milk quality and repeated mastitis is very important. While the impact of repeated mastitis within the same lactation has been investigated, the changes in body status from one lactation to the next, particularly concerning milk and blood metabolism, remain unclear.

Therefore, this study aims to investigate the influence of repeated mastitis on milk yield, milk composition, and metabolic status in the subsequent lactation. Obtaining results would facilitate understanding the pathophysiology of cows exposed to recurrent mastitis and identifying biomarkers that characterize such cows.

## 2 Materials and methods

### 2.1 Animals

This is a cohort study examining the future milk composition of individuals exposed to mastitis in previous lactation. Approximately 400 Holstein cows were used at the Zen-Noh Research Farm (Ibaraki, Japan). Cows were housed in a free-stall barn and fed Total Mixed Ration (TMR) *ad libitum* (allowing for a 10 % refusal). The TMR was formulated using NDS Professional ration formulation software (version3; RUM&N, NDS Professional, Reggio Nell’Emilia-Romagna, Italy) to provide an adequate amount of Metabolizable Energy and Metabolizable Protein for dairy cows day in milk (DIM) 80 producing 40 kg of milk according to National Research Council (2001) (Supplementary Table S1). Animals were milked two times a day at approximately 7:00 and 16:00.

### 2.2 Case definition

Cows with a positive California Mastitis Test (CMT), udder swelling, and selective treatment based on the bacterial culture of milk were classified as clinical mastitis. Repeated mastitis was defined as cows treated for mastitis and resumed lactation three or more times during the same lactation. No cases of bacterial infection spreading to other teats or quarter breasts after recovery from mastitis were observed. This study didn't consider subclinical mastitis derived solely from an increase in SCC.

### 2.3 Sample size and sample collection

From the 400 cows, based on treatment records provided by veterinarians, cows exposed to three or more episodes of clinical mastitis in the previous lactation were defined as repeated mastitis, and cows with no record of mastitis in the last two lactation periods were defined as healthy. Next, cows in the top 10 % of parity in the herd were excluded. Furthermore, cows started new lactation after dry-off and calving. They were confirmed free of clinical mastitis by the time of milk collection (DIM 60 to 90) by treatment records provided by the veterinarian, and SCC in the collected milk was confirmed to be less than 200,000 cells/ml. Finally, as the number of cows exposed to repeated mastitis was limited to 25, an equal number of individuals were randomly selected from the healthy cows. Thus, a total of 50 cows were used in this study.

Data on milk yield, fat, protein, and SCC for each cow in previous lactations were extracted from the Milk Recording Program in JAPAN (Livestock Improvement Association of Japan, INC.). Milk was collected equally from each quarter, and components and SCC in whole milk were measured using Milko Scan (Milko Scan CombiFoss™ FT+: Foss Japan, Tokyo). Blood samples were obtained before morning feeding using an EDTA-2Na tube from the tail vein and centrifuged (4 °C, 2000×*g*, 20 min).

### 2.4 Bacterial identification

After cleaning the teats, milk was manually collected in tubes, and 10 μl raw milk samples were seeded onto Easy media 4 (NIPPON ZENYAKU KOGYO Co., Ltd., Fukushima, Japan). Bacterial culture was conducted at 40 °C for 24–48 h, and the bacterial species was determined by visual observation based on the type of growth media and colony. If they cannot be identified on Easy Medium 4, raw milk samples were applied to BTB lactose agar and 5 % sheep blood agar medium and incubated at 33–37 °C for 18–24 h [25]. Isolated colonies were submitted to bacterial identification using MALDI-TOF MS at an external company (Biotyper, TechnoSuruga Laboratory Co., Ltd., Shizuoka, Japan). Results for bacterial species are presented in (Supplementary Table S2).

### 2.5 Untargeted metabolomic analysis

#### 2.5.1 Sample preparation

Untargeted metabolomics analysis was carried out using GC-MS (Gas chromatography-mass spectrometry), according to previously described [26].

In brief, acetonitrile, deionized water, and 1 mg/ml 2-isopropyl malic acid (internal standard) were added to the filtered skim milk sample and obtained aqueous solution. The samples were derivatized using a solid-phase derivatization kit (AiSTI SCIENCE Co., Ltd., Wakayama, Japan).

Methanol-chloroform-water (2.5:1:1) and 1 mg/m*l* 2-isopropylmalic acid (internal standard) were added to the filtered plasma sample and obtained aqueous solution was partially evaporated for 20 min and completely lyophilized. The lyophilized samples were derivatized by pyridine-containing methoxamine hydrochloride and N-methyl-N-trimethylsilyl-trifluoroacetamide treatment.

#### 2.5.2 GC-MS analysis

The derivatized milk sample (2 μl) or plasma sample (1 μl) was injected in spitless mode into GC-MS QP2020 NX (Shimadzu Corporation, Kyoto, Japan) and detailed parameter was previously described [26]. Metabolite identification was performed using the standard Smart Metabolites Database (Shimadzu Corporation), with each peak being automatically identified using free software MS-DIAL (version 4.80) and data analysis conducted using MetaboAnalyst (http://www.metaboanalyst.ca/).

### 2.6 Amino acid analysis

The free amino acid concentration was analyzed using an automated amino acid analyzer (L-8900; Hitachi High-Technologies Corporation, Tokyo) after each whole milk sample was deproteinized by 5 % trichloroacetic acid solution.

### 2.7 Statistical analysis

The metabolomics results were analyzed and visualized using MetaboAnalyst (https; //www.metaboanalyst.ca/). Partial least-squares discriminant analysis (PLS-DA) was used in metabolomics analysis to visualize the separation between healthy and recurrence mastitis cows and to identify essential metabolites responsible for the separation. GraphPad Prism 9 (GraphPad Software, San Diego, CA, USA) and R package 4.1.3 were used. Shapiro-Wilk test was performed to assess the variables with a normal distribution. Welch's *t*-test or Mann-Whitney's test was performed to determine the differences between groups. ANCOVA analysis set variables that included potential confounding factors (parity). *p* < 0.05 was considered statistically significant in all data.

The milk component prediction quality was investigated using Receiver Operating Characteristic (ROC) analysis, which included sensitivity, specificity, and area under the curve (AUC). R package 4.1.3 was used to perform ROC analysis. Sensitivity, Specificity, and Positive likelihood ratio (+LR) were calculated using the following formulas: Sensitivity = A/(A + C), Specificity = B/(B + D), +LR = Sensitivity/(1 – Specificity). In this formula, A represents the number of true positives, B represents the number of true negatives, C represents the number of false negatives, and D represents the number of false positives. Cohen's kappa coefficient was also used to confirm the reliability of the biomarker.

## 3 Results

### 3.1 Major milk parameters

Milk parameters, including milk yield, fat and protein concentration, and SCC during the previous lactation, were extracted from the monthly examination data of the Milk Recording Program (Table 1 and Supplementary Table S3). The collected milk samples from the subsequent lactation were analyzed using Milko Scan (Table 1 and Supplementary Table S4). The SCC count was significantly higher in cows that experienced three or more episodes of mastitis during the year, but no difference was observed in the subsequent lactation after dry-off. Fat concentrations were significantly lower in the next lactation, although no change was observed in the previous period when SCC levels differed (Table 1).

**Table 1:** Milk component of healthy (n = 25) and repeated mastitis cows (n = 25) during two lactation periods.

	Previous lactation (milk recording program)						Subsequent lactation (collected milk analysis)					
	Healthy (25)		Mastitis (25)				Healthy (25)		Mastitis (25)			
	Means	SE	Means	SE	*P-*value^1)^	*P*-value^2)^	Means	SE	Means	SE	*P*-value^1)^	*P*-value^2)^
Milk yield (kg/d)	36.52	1.05	35.80	1.07	0.64	0.56	36.01	1.83	32.13	2.26	0.19	0.20
Fat (%)	4.19	0.08	4.11	0.07	0.46	0.48	4.55	0.74	2.22	0.53	**<0.05**	**<0.05**
Protein (%)	3.30	0.04	3.20	0.05	0.13	0.14	3.29	0.10	3.36	0.09	0.55	0.57
SCC (x10^4^ cells/ml)	8.69	2.24	57.42	16.67	**<0.05**	**<0.05**	6.30	1.12	7.39	0.92	0.36	0.38
Parity	2.60	0.23	2.68	0.24	0.81	–	–	–	–	–		–

*P*-value^1)^ assessed by Independent sample WelchT test or Man-Whitney test according to Shapiro-wilk test.

*P*-value^2)^ assessed by ANCOVA analysis to considerate confounding factor of parity.

### 3.2 Milk metabolomic analysis

**Fig. 1:**
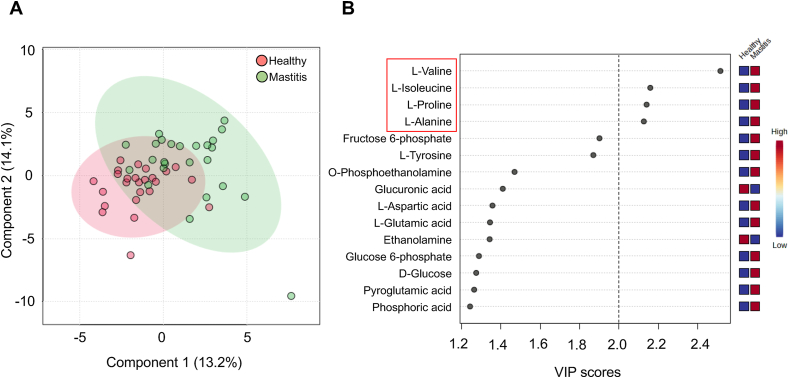
Difference in milk metabolites between healthy (n = 25) and repeated mastitis-experienced cows (n = 25). Cows with three or more episodes of mastitis in the previous lactation and cows with no history of mastitis were used, and milk samples were collected in the subsequent lactation after dry-off. A) PLS-DA score plots for an overall comparison of metabolite profiles of milk samples. B) Important features identified by PLS-DA and VIP scores express the metabolites' content relative to the color range. (For interpretation of the references to color in this figure legend, the reader is referred to the Web version of this article.)

Untargeted metabolomic analysis was performed on milk samples collected during the subsequent lactation to investigate changes in milk composition associated with repeated mastitis. A total of 68 metabolites were identified, and partial least-squares discriminant analysis (PLS-DA) revealed a distinct difference between the healthy and repeated mastitis groups (Fig. 1A). Variable importance for prediction (VIP) scores, based on PLS-DA, indicated that several metabolites, particularly free l-amino acids such as valine, isoleucine, proline, and alanine, were more abundant in the repeated mastitis group (VIP score value is more than 2.0) (Fig. 1B). l-glucuronic acid and ethanolamine were the abundant metabolites found in milk from the healthy group, but their VIP score values were low (less than 2.0).

### 3.3 Free l-amino acid concentration in milk and plasma

**Fig. 2:**
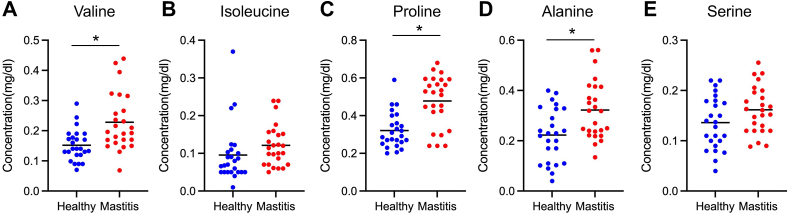
The levels of free amino acids in milk from healthy (n = 25) and repeated mastitis-experienced cows (n = 25) were compared, (A) valine, (B) isoleucine, (C) proline, (D) alanine, and (E) serine. Cows with three or more episodes of mastitis in the previous lactation and cows with no history of mastitis were used, and milk samples were collected in the subsequent lactation after dry-off. Statistical significance of *P < 0.05 by ANCOVA analysis.

**Fig. 3:**
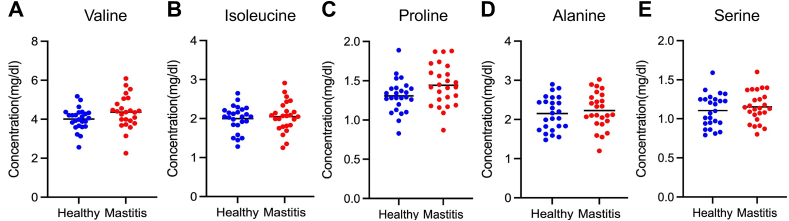
The levels of free amino acids in plasma from healthy (n = 25) and repeated mastitis-experienced cows (n = 25) were compared, (A) valine, (B) isoleucine, (C) proline, (D) alanine, and (E) serine. Cows with three or more episodes of mastitis in the previous lactation and cows with no history of mastitis were used, and blood samples were collected in the subsequent lactation after dry-off. Statistical significance of *P < 0.05 by ANCOVA analysis.

To confirm the increased abundance of free l-amino acids in milk, the concentration of these amino acids was measured using an automated amino acid analyzer. Consistent with the results from the untargeted metabolomic analysis, valine, proline, and alanine were significantly increased in the milk samples collected from cows that had experienced repeated mastitis in the last lactation (Fig. 2). However, no significant difference was observed in isoleucine and serine concentrations between the healthy and repeated mastitis groups. The concentration of free l-amino acids in plasma was also measured, but no significant difference in valine, proline, and alanine levels was found between the healthy and repeated mastitis cows (Fig. 3).

### 3.4 Biomarker analysis

To further demonstrate the association of free amino acids in milk with the experience of repeated mastitis, ROC curve analysis was performed (Table 2). A score of the area under the curve (AUC) greater than 0.7 indicates a good model for the ROC curve. Additionally, +LR (positive likelihood ratio) and Kappa coefficient values were provided to evaluate the model. A + LR value over 10 indicates a high effect on post-test probability [27]. Kappa values between 0.2 and 0.4 were considered fair, 0.4 to 0.6 moderate, 0.6 to 0.8 substantial, and >0.8 excellent agreement [28]. Each valine and proline showed a moderate biomarker potential based on Kappa coefficients. Furthermore, the combination of valine and proline as biomarkers provided the highest specificity (100 %), +LR (infinity), and substantial biomarker potential based on Kappa coefficients (0.68).

**Table 2:** Assessment models to estimate past repeated mastitis experiences.

	Valine	Isoleucine	Proline	Alanine	Valine + Proline
Threshold	0.145 mg/dl	0.095 mg/dl	0.415 mg/dl	0.235 mg/dl	(0.145 mg/dl+0.415 mg/dl)
AUC	**0.78**	**0.71**	**0.81**	**0.72**	–
Sensitivity	0.92	0.68	0.76	0.76	0.68
Specificity	0.56	0.68	0.84	0.56	1.00
+LR	2.30	2.13	4.75	1.73	**Infinity**
Kappa coefficients	**0.48**	0.36	**0.60**	0.32	**0.68**

AUC was considered as more than 0.7 is a good model for the ROC curve.

Sensitivity: the ability of a test to correctly identify repeated mastitis cow.

Specificity: the ability of a test to correctly identify a healthy cow.

+LR value over 10 indicates a high effect on post-test probability..

Kappa values between 0.2 and 0.4 were considered fair, 0.4 to 0.6 moderate, 0.6 to 0.8 substantial, and >0.8 excellent agreement.

## 4 Discussion

Mastitis is a prevalent and economically significant disease in dairy cows, leading to reduced milk production and quality. Repeated episodes of mastitis within a single lactation can have detrimental effects, increasing the risk of culling and persistent milk losses [21]. It has long been hypothesized that repeated mastitis episodes can change milk production ability in subsequent lactations, resulting in reduced milk productivity. However, the changes in metabolic status, such as milk metabolites, are still unclear. To the best of our knowledge, no previous studies have investigated the changes in milk components and metabolites in the subsequent lactation between healthy and repeated mastitis in the past. This study aimed to fill this knowledge gap and demonstrate the difference in milk yield, milk composition, and metabolic status in the subsequent lactation between healthy and repeated mastitis in the previous lactation through the cohort study. Specifically, we focused on the concentrations of certain free l-amino acids, namely valine, proline, and alanine, which we hypothesized to be altered in milk exposed to repeated mastitis compared to healthy milk, irrespective of somatic cell count (SCC). Additionally, we developed assessment models utilizing the valine and proline concentrations in milk to characterize cows exposed to repeated mastitis.

During the subsequent lactation period, there was a significant decrease in milk fat concentration in cows that had experienced repeated mastitis. This decline in milk fat concentration suggests that cows exposed to recurrent mastitis may have reduced milk production capacity in the subsequent lactation due to the effects of repeated infections. Consistent with our findings, previous studies have demonstrated that once a cow contracts mastitis, it never fully recovers its earlier production levels [[29], [30], [31], [32]]. The change in milk yield associated with mastitis is often influenced by the types of bacteria that invade the mammary gland's secretory epithelial cells [33]. The lower fat percentage in mastitis milk can be attributed to lipolysis, the host's nonspecific immune response to invading bacteria. This can be explained by the inflammatory reactions that reduce mammary epithelial cells' synthetic and secretory capacity [34].

It has been documented in previous studies that milk samples exposed to clinical mastitis exhibit elevated concentrations of free amino acids, such as valine and proline [19,35]. This increase in amino acids is typically attributed to bacterial fermentation and protein degradation, and it is closely linked to the somatic cell count (SCC) [[36], [37], [38]]. However, an intriguing observation in our study was that high concentrations of amino acids were detected in the milk samples collected from cows that had experienced repeated mastitis in the previous lactation despite having average SCC in the subsequent lactation. This finding suggests that persistent or repeated bacterial infections may impact the properties of mammary epithelial cells, leading to alterations in amino acid metabolism in milk, which are caused by changes in subsequent lactation. A recent study demonstrated that treatment with IFN-γ, an immune-regulatory cytokine, in bovine mammary gland epithelial cells, resulted in significant changes in metabolite profiles, including arginine and proline metabolism [39]. Further investigations are warranted to elucidate the underlying mechanisms of repeated infections and/or caused changes in mammary gland tissue.

A significant proportion of cows that experience clinical mastitis during lactation develop additional mastitis episodes within the same lactation [40]. It has been observed that cows with a history of mastitis are more prone to new infections, irrespective of the pathogen involved [41]. The recurrence of mastitis can also be attributed to persistent infection, where the infection persists even after the resolution of clinical signs, leading to subsequent flare-ups of mastitis [42]. A clinical mastitis event in the previous lactation has also been identified as a risk factor for mastitis recurrence in the subsequent lactation [[43], [44], [45], [46]]. These observations indicate that cows with repeated episodes of mastitis in the same lactation are at a higher risk of developing mastitis in the subsequent lactation. In our preliminary data, the percentage of cows culled in the subsequent lactation that had been mastitis-free for the previous two years was 14 %, whereas the percentage that had experienced three or more episodes of mastitis in the last lactation was 37 % (data not shown). Although the cause of culling in these cases is unknown, it is crucial to identify cows exposed to repeated mastitis to make informed cow management decisions in subsequent lactations.

Numerous diagnostic tools and biomarkers were available during that period to identify clinical and subclinical mastitis, including milk SCC, the California Mastitis Test (CMT), low molecular weight metabolites, and udder surface temperature [16,19,35,[47], [48], [49]]. This study aims to provide assessment models for characterizing cows that have experienced repeated mastitis, utilizing valine and proline concentrations in milk. The proposed model represents a novel approach to assessing the risk of mastitis by identifying individuals who have previously developed mastitis, irrespective of high SCC or other mastitis symptoms. These findings significantly contribute to understanding mastitis's pathobiology and etiology. Additionally, previous reports have indicated that serum amino acids, including valine and proline, are suitable biomarkers for subclinical mastitis [18,20]. Therefore, these amino acids are crucial in distinguishing healthy cows from those exposed to mastitis. With further studies, they can be utilized as screening biomarkers for future mastitis incidence.

One of the study's limitations was the number of herds and cows. According to the reasonable power of 0.8 and moderate effect size f = 0.25 proposed by Cohen [50], the sample size required for this study was calculated using Gpower3.1, which initially required 64 n animals per group. Future studies should be conducted using many herds and cows to verify the difference in milk composition and metabolic status in the subsequent lactation after dry-off. Another limitation of the study is the lack of detailed information about individual cow status, such as a short time record of the number of SCC (incidence of subclinical mastitis), udder condition, genetic background, and difficulty adjusting the parity and time of sample collection for the cows. Confounding factors associated with milk production properties such as parity, age, calving interval, and herd were considered to affect milk production ability relatively [51,52]. In this study, only the parity was included, but the other confounding factor might have needed to be noticed. In addition, this study can't rule out the possibility that a healthy herd contains a mixture of subclinical mastitis individuals who are CMT-negative and have no clinical symptoms. However, we believe that the results obtained in this study by defining individuals exposed to repeated mastitis with clinical symptoms as recurrent cows and comparing them with the herd defined as healthy are well worthwhile.

## 5 Conclusion

Our results propose new insights into the pathogenesis and etiology of mastitis, such as that repeated cases alter milk components, amino acids, and other metabolites during the subsequent lactation period. In addition, a new biomarker that detects cows with repeated mastitis has been discovered because it can be utilized as a screening biomarker for future mastitis incidence. These findings will help dairy farmers develop the following strategies to prevent significant economic losses caused by mastitis.

## Ethics statement

The animal experiments were approved by the Animal Care Committee of the ZEN-NOH Central Research Institute for Feed and Livestock (No. 1601-017).

## Funding

This study was supported by the Livestock Promotional Subsidies of the 10.13039/100015103Japan Racing Association (JRA) , Japan (#2022-36, K.N.).

## Data availability statement

Data will be made available on request.

## CRediT authorship contribution statement

**Shiho Miyata:** Writing – original draft, Investigation, Formal analysis, Data curation, Conceptualization. **Lijie Fan:** Writing – review & editing, Investigation, Formal analysis. **Jun Kambe:** Writing – review & editing, Investigation, Formal analysis. **Mohammad Ibrahim Qasimi:** Writing – review & editing, Writing – original draft. **Satoshi Takemoto:** Writing – review & editing, Investigation, Formal analysis. **Masahiko Ito:** Writing – review & editing, Investigation. **Chunmei Li:** Writing – review & editing, Formal analysis. **Yuki Yamamoto:** Writing – review & editing, Investigation, Formal analysis. **Kentaro Nagaoka:** Writing – review & editing, Project administration, Data curation, Conceptualization.

## Declaration of competing interest

The authors declare that they have no known competing financial interests or personal relationships that could have appeared to influence the work reported in this paper.
